# Long-Term Exposure to Ambient Air Pollution and Incidence of Cerebrovascular Events: Results from 11 European Cohorts within the ESCAPE Project

**DOI:** 10.1289/ehp.1307301

**Published:** 2014-05-16

**Authors:** Massimo Stafoggia, Giulia Cesaroni, Annette Peters, Zorana J. Andersen, Chiara Badaloni, Rob Beelen, Barbara Caracciolo, Josef Cyrys, Ulf de Faire, Kees de Hoogh, Kirsten T. Eriksen, Laura Fratiglioni, Claudia Galassi, Bruna Gigante, Aki S. Havulinna, Frauke Hennig, Agneta Hilding, Gerard Hoek, Barbara Hoffmann, Danny Houthuijs, Michal Korek, Timo Lanki, Karin Leander, Patrik K. Magnusson, Christa Meisinger, Enrica Migliore, Kim Overvad, Claes-Göran Östenson, Nancy L. Pedersen, Juha Pekkanen, Johanna Penell, Goran Pershagen, Noreen Pundt, Andrei Pyko, Ole Raaschou-Nielsen, Andrea Ranzi, Fulvio Ricceri, Carlotta Sacerdote, Wim J.R. Swart, Anu W. Turunen, Paolo Vineis, Christian Weimar, Gudrun Weinmayr, Kathrin Wolf, Bert Brunekreef, Francesco Forastiere

**Affiliations:** 1 Department of Epidemiology, Lazio Regional Health Service, Rome, Italy; 2 Institute of Epidemiology II, Helmholtz Zentrum München, German Research Center for Environmental Health, Neuherberg, Germany; 3 Danish Cancer Society Research Center, Danish Cancer Society, Copenhagen, Denmark; 4 Center for Epidemiology and Screening, Department of Public Health, University of Copenhagen, Copenhagen, Denmark; 5 Institute for Risk Assessment Sciences, Utrecht University, Utrecht, the Netherlands; 6 Department of Neurobiology, Care Sciences and Society, Aging Research Center, Karolinska Institutet, Stockholm, Sweden; 7 Environment Science Center, University of Augsburg, Augsburg, Germany; 8 Institute of Environmental Medicine, Karolinska Institutet, Stockholm, Sweden; 9 MRC-PHE Centre for Environment and Health, Department of Epidemiology and Biostatistics, Imperial College London, London, United Kingdom; 10 Stockholm Gerontology Research Center, Stockholm, Sweden; 11 Division of Clinical Geriatrics, Karolinska University Hospital, Stockholm, Sweden; 12 Unit of Cancer Epidemiology, “Città della Salute e della Scienza” Hospital-University of Turin, and Center for Cancer Prevention Piemonte, Turin, Italy; 13 Department of Chronic Disease Prevention, National Institute for Health and Welfare, Helsinki, Finland; 14 IUF-Leibniz Research Institute for Environmental Medicine, Düsseldorf, Germany; 15 Department of Molecular Medicine and Surgery, Karolinska Institutet, Karolinska University Hospital, Stockholm, Sweden; 16 Medical Faculty, University of Düsseldorf, Düsseldorf, Germany; 17 National Institute for Public Health and the Environment, Bilthoven, the Netherlands; 18 Department of Environmental Health, National Institute for Health and Welfare, Kuopio, Finland; 19 Department of Medical Epidemiology and Biostatistics, Karolinska Institutet, Stockholm, Sweden; 20 MONICA/KORA Myocardial Infarction Registry, Central Hospital of Augsburg, Augsburg, Germany; 21 Section for Epidemiology, Department of Public Health, Aarhus University, Aarhus, Denmark; 22 Department of Cardiology, Aalborg University Hospital, Aalborg, Denmark; 23 Institute for Medical Informatics, Biometry and Epidemiology, University Hospital of Essen, Essen, Germany; 24 Environmental Health Reference Centre, Regional Agency for Environmental Prevention of Emilia-Romagna, Modena, Italy; 25 Molecular and Genetic Epidemiology Unit, Human Genetics Foundation, Turin, Italy; 26 Department of Neurology, University of Duisburg-Essen, Essen, Germany; 27 Institute of Epidemiology and Medical Biometry, Ulm University, Ulm, Germany; 28 Department of Epidemiology, Julius Center for Health Sciences and Primary Care, University Medical Center Utrecht, Utrecht, the Netherlands

## Abstract

Background: Few studies have investigated effects of air pollution on the incidence of cerebrovascular events.

Objectives: We assessed the association between long-term exposure to multiple air pollutants and the incidence of stroke in European cohorts.

Methods: Data from 11 cohorts were collected, and occurrence of a first stroke was evaluated. Individual air pollution exposures were predicted from land-use regression models developed within the European Study of Cohorts for Air Pollution Effects (ESCAPE). The exposures were: PM_2.5_ [particulate matter (PM) ≤ 2.5 μm in diameter], coarse PM (PM between 2.5 and 10 μm), PM_10_ (PM ≤ 10 μm), PM_2.5_ absorbance, nitrogen oxides, and two traffic indicators. Cohort-specific analyses were conducted using Cox proportional hazards models. Random-effects meta-analysis was used for pooled effect estimation.

Results: A total of 99,446 study participants were included, 3,086 of whom developed stroke. A 5-μg/m^3^ increase in annual PM_2.5_ exposure was associated with 19% increased risk of incident stroke [hazard ratio (HR) = 1.19, 95% CI: 0.88, 1.62]. Similar findings were obtained for PM_10_. The results were robust to adjustment for an extensive list of cardiovascular risk factors and noise coexposure. The association with PM_2.5_ was apparent among those ≥ 60 years of age (HR = 1.40, 95% CI: 1.05, 1.87), among never-smokers (HR = 1.74, 95% CI: 1.06, 2.88), and among participants with PM_2.5_ exposure < 25 μg/m^3^ (HR = 1.33, 95% CI: 1.01, 1.77).

Conclusions: We found suggestive evidence of an association between fine particles and incidence of cerebrovascular events in Europe, even at lower concentrations than set by the current air quality limit value.

Citation: Stafoggia M, Cesaroni G, Peters A, Andersen ZJ, Badaloni C, Beelen R, Caracciolo B, Cyrys J, de Faire U, de Hoogh K, Eriksen KT, Fratiglioni L, Galassi C, Gigante B, Havulinna AS, Hennig F, Hilding A, Hoek G, Hoffmann B, Houthuijs D, Korek M, Lanki T, Leander K, Magnusson PK, Meisinger C, Migliore E, Overvad K, Östenson CG, Pedersen NL, Pekkanen J, Penell J, Pershagen G, Pundt N, Pyko A, Raaschou-Nielsen O, Ranzi A, Ricceri F, Sacerdote C, Swart WJ, Turunen AW, Vineis P, Weimar C, Weinmayr G, Wolf K, Brunekreef B, Forastiere F. 2014. Long-term exposure to ambient air pollution and incidence of cerebrovascular events: results from 11 European cohorts within the ESCAPE project. Environ Health Perspect 122:919–925; http://dx.doi.org/10.1289/ehp.1307301

## Introduction

Air quality standards are under revision in Europe, and a new policy is due from the European Parliament. As part of this process, the European Union (EU) has indicated several specific issues of concern when considering the chronic effects of long-term exposure to ambient air pollution, especially the effects of fine particulate matter (PM with aerodynamic diameter ≤ 2.5 μm; PM_2.5_) on cardiovascular and respiratory health in Europe.

Substantial evidence from large studies conducted in the United States (Krewski et al. 2009; Laden et al. 2006; Miller et al. 2007) and Canada (Crouse et al. 2012) has documented effects of fine particles on natural and cardiopulmonary mortality as the primary end points. Only a limited number of studies have been conducted in Europe (Andersen et al. 2012; Atkinson et al. 2013; Brunekreef et al. 2009; Filleul et al. 2005; Gehring et al. 2006), most including only one cohort from a single country, and focusing on the intracohort spatial contrasts rather than on differences across study areas. It is therefore uncertain to what degree the results can be generalized to other areas in Europe. In recent years, some attempts have also been made to investigate the relationship between long-term air pollution exposure and incidence of cerebrovascular disease, providing conflicting evidence (Andersen et al. 2012; Atkinson et al. 2013; Krewski et al. 2009; Miller et al. 2007). Recently, Maaten and Brook (2011) indicated that the relationship “merits further attention on global research and public policy agendas.”

Biological mechanisms linking long-term air pollution exposure to chronic damage of the cardiovascular system may include endothelial dysfunction and vasoconstriction, increased blood pressure, prothrombotic and coagulant changes, systemic inflammatory and oxidative stress responses, autonomic imbalance and arrhythmias, and the progression of atherosclerosis. On these bases, the American Heart Association delivered a scientific statement concluding that the overall evidence is consistent with PM playing a causal role in cardiac morbidity and mortality (Brook et al. 2010). For cerebrovascular diseases, several studies have indicated the effects of short-term exposures potentially leading to ischemic stroke (O’Donnell et al. 2011; Wellenius et al. 2012). However, the evidence of a link between long-term exposure to air pollution and cerebrovascular events is less developed.

The European Study of Cohorts for Air Pollution Effects (ESCAPE) project was designed to assess the long-term exposure of the population to air pollution and to investigate exposure–response relationships and thresholds for a number of adverse health outcomes (ESCAPE 2007). Our objective was to estimate the association between long-term exposure to ambient air pollution, especially PM mass, black carbon, and nitrogen oxides, and the incidence of stroke in 11 European cohorts. A companion paper focusing on incident coronary events has been recently published (Cesaroni et al. 2014).

## Methods

*Study population*. Individual data were collected for 11 existing cohort studies from Finland, Sweden, Denmark, Germany, and Italy. Individuals had been enrolled at different periods, ranging from 1992 to 2007, and were followed until migration, death, or the occurrence of the study outcome until 2006–2010. Baseline individual data included sociodemographic characteristics (age, sex, marital status, education, occupation), lifestyle variables (smoking status, smoking intensity and duration, physical activity, alcohol consumption), physiological parameters [body mass index (BMI), cholesterol level], chronic conditions (diabetes, hypertension), and modeled road traffic noise exposure at the residential address. In addition, different area-level socioeconomic variables were collected for each cohort. Finally, if the study area included different degrees of urbanization, a binary “rural” indicator was used to characterize each residential address. (For futher details, see Supplemental Material, Table S1.)

The original cohort studies were approved by appropriate institutional medical ethics committees and undertaken in accordance with the Declaration of Helsinki (http://www.wma.net/en/30publications/10policies/b3/). Each cohort study followed the rules for ethics and data protection set up in the country in which they were based.

*Outcome definition*. The identification of first cerebrovascular events during follow-up was accomplished by interview, inspection of medical records and death certificates, or by record linkage with mortality registries and hospital discharge databases. Prevalent cases of either coronary or cerebrovascular disease at baseline were excluded. Methods to define and ascertain prevalent cases differed between the cohorts, as reported in Supplemental Material (“Methods,” pp. 3–13).

*Exposure assessment*. Long-term exposure to ambient air pollutants at the residential address of each individual was estimated following a three-step procedure. First, PM_2.5_, PM_2.5_ absorbance, PM_10_ (aerodynamic diameter ≤ 10 μm), nitrogen dioxide (NO_2_), and nitrogen oxides (NO_x_) were measured between October 2008 and April 2011 using standardized protocols (Cyrys et al. 2012; Eeftens et al. 2012b). Coarse PM was calculated as the difference between PM_10_ and PM_2.5_. Second, land-use regression (LUR) models were developed for each study area and pollutant (Beelen et al. 2013a; Eeftens et al. 2012a). Third, individual annual exposures were predicted using these models. In addition, traffic intensity on the nearest road (vehicles per day), and traffic load on major roads within a 100-m buffer (product of traffic intensity and length of roads intersecting the buffer) were computed. Noise exposure was assessed locally by calculating the day-evening-night equivalent noise level (*L*_den_) for the most exposed façade of dwellings (see Supplemental Material, “Methods,” pp. 3–13).

*Statistical analysis*. We carried out the analyses using a two-stage approach, with cohort-specific analyses in the first stage and random-effects meta-analysis in the second.

At the first stage, we fitted Cox proportional hazards regression models in each cohort, with age as the underlying time variable. All analyses were conducted using a common statistical protocol and STATA script (Stata software, version 11; StataCorp, College Station, TX, USA). We defined adjustment models *a priori*. We used three degrees of adjustment: *a*) estimates adjusted only for sex and calendar year of enrollment (model 1); *b*) adjustment for the shared set of potential individual-level confounders: sex, calendar year, marital status, education, occupational status, smoking status, smoking duration among ever smokers, and smoking intensity among current smokers (model 2); and *c*) adjustment for the shared set of individual-level confounders (model 2) plus one cohort-specific area-level socioeconomic variable (model 3, also referred to as the “main” model). All confounders were baseline characteristics and were included as fixed covariates in the regression models. Only study participants with no missing information from any of the exposures and confounders in the main model were included in all analyses.

We performed a number of additional analyses within each cohort:

We addressed the potential effect due to lack of adjustment for relevant cardiovascular risk factors. With this aim, *a*) we adjusted for intermediate variables only (diabetes and hypertension, available in all the cohorts); *b*) we adjusted for cardiovascular confounders available in most cohorts [physical activity, alcohol consumption and BMI (available in eight cohorts)]; and *c*) we added the cholesterol level (available in four cohorts).
We added the “rural” indicator to the main model to better account for different degrees of urbanization within the study areas.
We evaluated potential confounding by noise.
We restricted the analyses to people who never changed address during follow-up.
We performed diagnostic tools to check the proportionality-hazard (PH) assumption for the categorical predictors in the main model, and stratified the Cox model for the predictors that did not meet the PH assumption.
We evaluated the potential for spatial clustering by running “frailty” models (Jerrett et al. 2003).
We evaluated the robustness of the results by excluding the most influential cohort [the Diet, Cancer and Health cohort (DCH)] and by stratifying the cohorts by performance of the LUR model, choosing a cut-off point of 0.6 for the leave-one-out cross-validation (LOOCV) *R^2^* coefficient (Eeftens et al. 2012a).

Next, we evaluated a number of individual characteristics considered *a priori* as potential effect modifiers: sex, age during follow-up (< 60 years, 60–74 years, ≥ 75 years), education, smoking status, BMI (< 25 kg/m^2^, 25–29 kg/m^2^, ≥ 30 kg/m^2^), previous diabetes or hypertension, and residence in rural/urban area.

Finally, we examined in each cohort the shape of the relationship between each exposure and the study outcome by *a*) inputting the exposure term as a natural cubic spline with three equally-spaced inner knots, and comparing the model fit of the linear and the spline models, via likelihood-ratio test; and *b*) implementing “threshold models,” in which threshold concentrations were defined *a priori* for each exposure, and cohort-specific models were run only on observations with predicted exposures below each threshold in turn.

In the second stage of the analysis, we pooled the cohort-specific results by random-effects meta-analysis (DerSimonian and Laird 1986). We evaluated the presence of heterogeneity in the cohort-specific results by applying the chi-square test from Cochran’s *Q* statistic, which was then quantified by calculating the *I*^2^ statistic (Higgins and Thompson 2002). We considered cohort-specific effect estimates to be significantly heterogeneous when *I*^2^ was > 50% or the *p*-value of the chi-square test was < 0.05. Finally, we checked the presence of effect modification across strata of each modifier by meta-analyzing the pooled estimates from the different strata, and by performing the chi-square test of heterogeneity. We considered pooled strata-specific effect estimates to be significantly different when the *p*-value of the chi-square test was < 0.10.

We expressed all results as hazard ratios (HRs), and 95% confidence intervals (CIs), relative to fixed increments in each exposure, defined *a priori*: 5 μg/m^3^ for PM_2.5_ and coarse PM, 10 μg/m^3^ for PM_10_ and NO_2_, 20 μg/m^3^ for NO_x_, 10^–5^/m for PM_2.5_ absorbance, 5,000 motor vehicles/day for traffic intensity on the nearest road, and 4,000,000 motor vehicles × meters per day.

All first-stage and meta-analyses were fit using the Stata software, version 11 (StataCorp). The frailty and spline models were fit using R software, version 2.15.0 (R Project for Statistical Computing; http://R-project.org).

## Results

A total of 111,931 participants were under study. After the exclusion of the prevalent cases and of participants with missing exposure, a total of 105,025 participants remained. However, 5,579 participants had missing information on any of the variables in the main model; therefore, 99,446 participants (88.8% of the original study population, and 92.4% of the original cohorts after exclusion of prevalent cases) were included in the analyses, providing ≥ 1 million person-years of observation. 3,086 incident stroke events were registered during the follow-up. The majority of the stroke cases with defined etiology were coded as ischemic stroke; however, 43% of all cases were undefined, thus precluding the possibility of analyzing different types of stroke separately. The baseline age distribution was heterogeneous across cohorts, with mean values ranging from 44 years (two Italian cohorts) to 74 years (a Swedish cohort), whereas sex, education, and occupation had less variability. The percentage of current smokers at baseline was the highest in southern Europe and the lowest in Sweden and Germany (Table 1). The comparison of the studied population before and after the exclusion of the participants with missing data on the confounders did not show differences in relation to air pollution exposure (i.e., PM_2.5_) and occurrence of the study outcome.

**Table 1:** Study population: individual baseline characteristics, 11 cohorts.

Variables	FINRISK	SNAC-K	SALT	60y	SDPP	DCH	HNR	KORA	EPIC- Turin	SIDRIA- Turin	SIDRIA- Rome
Participants (*n*)	9,995	2,684	6,084	3,686	7,723	35,693	4,433	7,581	7,230	5,137	9,200
Person-years at risk	105,060	16,256	51,756	39,978	106,995	464,055	34,941	76,027	91,490	56,366	102,894
Percent of the original cohort^*a*^	89.3	79.8	86.4	87.1	97.2	90.5	92.1	83.2	82.4	95.1	86.8
Cases (*n*)	184	164	216	125	107	1,848	71	210	55	37	69
Years of enrollment	1992, 1997, 2002, 2007	2001–2004	1998–2002	1997–1999	1992–1998	1993–1997	2000–2003	1994–1995, 1999–2001	1993–1998	1999	1999
Individual characteristics
Age, years [mean (minimum–maximum)]	48 (25–74)	74 (60–102)	59 (42–97)	60 (59–61)	47 (35–56)	57 (50–66)	59 (45–75)	50 (25–82)	50 (35–67)	44 (27–76)	44 (28–63)
Sex, female (%)	55	65	58	53	61	54	52	51	48	52	53
Marital status (%)
Single	16	15	14	5	17^*b*^	7	6	10	6	2	0
Married/living with partner	70	47	67	71	83	69	75	77	86	95	100
Divorced/separated	11	13	11	17	—	18	10	7	5	1	0
Widowed	3	25	8	7	—	6	9	6	3	2	0
Education (%)
≤ Primary school	30	27	21	28	26	30	11	12	44	17	45
≤ Secondary school or equivalent	52	42	43	44	45	47	56	75	43	71	40
≥ University degree	17	31	36	28	29	23	33	13	14	11	15
Occupational status (%)
Employed/self-employed	71	75	—	51	92	80	42	60	—	73	71
Unemployed	6	25^*c*^	—	10	8^*c*^	20^*c*^	6	3	—	7	4
Homemaker/housewife	4	—	—	8	—	—	14	14	—	21	25
Retired	19	—	—	31	—	—	38	23	—	0	0
Smoking status (%)
Current smoker	26	15	23	21	26	36	23	25	24	41	42
Former smoker	28	34	43	38	36	28	33	31	33	21	23
Never-smoker	46	51	35	40	37	36	43	44	43	38	34
Years of smoking, among ever smokers (mean ± SD)	15 ± 12	30 ± 17	—	26 ± 13	20 ± 10	29 ± 10	36 ± 9^*d*^	21 ± 12	23 ± 10	18 ± 8	18 ± 7
No. of cigarettes/day, among current smokers (mean ± SD)	15 ± 9	11 ± 8	13 ± 8	13 ± 7	14 ± 7	17 ± 10	17 ± 12	15 ± 11	14 ± 9	15 ± 9	15 ± 9
Abbreviations: DCH, Danish Diet, Cancer and Health cohort study; EPIC, European Prospective Investigation into Cancer and Nutrition; FINRISK, Finland Cardiovascular Risk Study; HNR, Heinz Nixdorf Recall Study; KORA, Cooperative Health Research in the Augsburg Region; SALT, Screening Across the Lifespan Twin study; SDPP, Stockholm Diabetes Prevention Program study; SIDRIA, International Study on Asthma and Allergies in Childhood; SNAC-K, Swedish National Study on Aging and Care in Kungsholmen; 60y, 60-year-olds study. ^***a***^After exclusion of prevalent cases and observations with missing information on any of the variables in the base model. ^***b***^All except married/living with partner. ^***c***^All except employed. ^***d***^Only among current smokers.											

A map of the study areas and further details on individual and area-level characteristics are available in Supplemental Material, Figure S1 and Table S1. Exposure levels and ranges were generally higher in Italy than in the other areas (Table 2). More details on air pollution exposures are reported in the Supplemental Material, Tables S2 and S3.

**Table 2:** Study population: environmental exposures at residential address, 11 cohorts.

Exposure	FINRISK	SNAC-K	SALT	60y	SDPP	DCH	HNR	KORA	EPIC- Turin	SIDRIA- Turin	SIDRIA- Rome
Environmental exposures at residential address
PM_2.5_ (μg/m^3^)	8 (6–9)	8 (6–10)	7 (5–9)	7 (5–9)	7 (5–8)	11 (10–13)	18 (17–20)	14 (12–15)	30 (27–33)	31 (29–34)	19 (17–23)
Coarse PM (μg/m^3^)	7 (4–11)	8 (1–19)	7 (2–12)	7 (1–12)	6 (1–9)	6 (4–7)	10 (7–12)	6 (5–8)	16 (12–20)	17 (13–20)	17 (12–24)
PM_10_ (μg/m^3^)	14 (10–20)	16 (6–29)	15 (7–21)	15 (7–21)	14 (6–17)	17 (14–20)	28 (25–32)	20 (16–24)	46 (39–52)	48 (41–54)	36 (31–47)
PM_2.5_ absorbance (10^–5^/m)	0.9 (0.5–1.2)	0.8 (0.5–1.2)	0.6 (0.4–0.9)	0.6 (0.4–0.9)	0.5 (0.4–0.7)	1.2 (0.8–1.5)	1.6 (1.2–2.2)	1.7 (1.5–2.0)	3.1 (2.3–3.6)	3.2 (2.6–3.8)	2.7 (2.2–4.0)
NO_2_ (μg/m^3^)	15 (9–24)	17 (9–25)	11 (7–20)	11 (6–20)	8 (6–11)	16 (8–30)	30 (23–39)	19 (14–26)	53 (34–68)	60 (42–77)	39 (26–56)
NO_x_ (μg/m^3^)	24 (14–41)	33 (15–58)	19 (12–40)	19 (12–39)	14 (12–20)	27 (7–66)	51 (33–72)	32 (24–47)	96 (62–132)	107 (79–162)	82 (39–122)
Background NO_2_ (μg/m^3^)	15 (10–19)	16 (12–19)	11 (6–17)	10 (5–17)	7 (4–10)	14 (8–20)	26 (24–30)	18 (14–24)	39 (27–45)	40 (33–45)	41 (29–53)
Daily no. of vehicles on the nearest road	1,670 (50–9,011)	3,726 (500–21,828)	1,454 (500–6,000)	1,455 (500–6,300)	864 (500–2,575)	2,994 (200–16,145)	—	1,613 (500–8,367)	3,907 (0–23,951)	4,290 (0–24,379)	2,966 (500–15,312)
Total traffic load (intensity*length) on major roads in a 100-m buffer (thousands)	633 (0–3,711)	2,307 (0–6,572)	578 (0–3,437)	521 (0–3,048)	109 (0–986)	1,274 (51–4,719)	1,017 (0–4,302)	438 (0–2,790)	466 (0–2,340)	804 (0–4,197)	1,417 (0–6,947)
Pearson correlation between PM_2.5_ and
PM_10_	0.67	0.70	0.49	0.50	0.31	0.74	0.90	0.42	0.62	0.56	0.92
Coarse PM	0.10	0.71	0.50	0.50	0.32	0.60	0.51	0.38	0.51	0.32	0.90
PM_2.5_ absorbance	0.98	0.98	0.84	0.84	0.90	0.49	0.76	0.50	0.77	0.73	0.78
NO_2_	0.41	0.82	0.60	0.61	0.61	0.57	0.63	0.45	0.72	0.67	0.69
Abbreviations: EPIC, European Prospective Investigation into Cancer and Nutrition; DCH, Danish Diet, Cancer and Health cohort study; FINRISK, Finland Cardiovascular Risk Study; HNR, Heinz Nixdorf Recall Study; KORA, Cooperative Health Research in the Augsburg Region; SALT, Screening Across the Lifespan Twin study; SDPP, Stockholm Diabetes Prevention Program study; SIDRIA, International Study on Asthma and Allergies in Childhood; SNAC-K, Swedish National Study on Aging and Care in Kungsholmen; 60y, 60-year-olds study. Data are expressed as means (5th–95th percentile ranges) or as Pearson correlation coefficients.											

Table 3 shows the HRs (95% CIs) from models 1, 2, and 3 (the main model) for all pollutants and traffic variables. Estimates were the highest in the first model, and significantly heterogeneous for most exposures. Estimates and heterogeneity decreased when adjusting for the common set of individual-level and area-level confounders, however heterogeneity remained in the main model for PM_2.5_. None of the associations was statistically significant. The highest estimate was found with PM_2.5_: A 5-μg/m^3^ increase in PM_2.5_ was associated with a 19% increased risk of incident stroke (HR = 1.19; 95% CI: 0.88, 1.62; *I*^2^ = 49%). PM_2.5_ and PM_10_ cohort-specific and pooled results are reported in Supplemental Material, Figure S2.

**Table 3:** Association between air pollution exposures and stroke incidence in the 11 cohorts under study.

Exposure	Fixed increase	Cohorts (*n*)	Participants (*n*)	Model 1^*a*^ [HR (95% CI)]	Model 2^*b*^ [HR (95% CI)]	Model 3^*c*^ [HR (95% CI)]
PM_2.5_	5 μg/m^3^	11	99,446	1.26 (0.92, 1.71)*	1.16 (0.88, 1.53)	1.19 (0.88, 1.62)*
Coarse PM	5 μg/m^3^	11	99,446	1.07 (0.92, 1.24)	1.02 (0.89, 1.18)	1.02 (0.90, 1.16)
PM_10_	10 μg/m^3^	11	99,446	1.15 (0.91, 1.46)*	1.11 (0.90, 1.36)	1.11 (0.90, 1.36)
PM_2.5_ absorbance	10^–5^/m	11	99,446	1.17 (0.86, 1.59)*	1.08 (0.82, 1.42)	1.08 (0.83, 1.41)
NO_2_	10 μg/m^3^	11	99,446	1.04 (0.91, 1.19)*	1.00 (0.88, 1.14)*	0.99 (0.89, 1.11)
NO_x_	20 μg/m^3^	11	99,446	1.04 (0.94, 1.16)	1.01 (0.91, 1.12)	0.98 (0.89, 1.07)
Traffic intensity on the nearest road	5,000 mv/day	10^*d*^	95,013	1.00 (0.97, 1.02)	0.99 (0.97, 1.02)	0.99 (0.97, 1.02)
Traffic load on major roads in a 100-m buffer	4,000,000 mv/day*m	11	99,446	1.05 (0.97, 1.13)	1.02 (0.95, 1.10)	1.02 (0.95, 1.10)
mv, Motor vehicles. ^***a***^Adjusted for age, year of enrollment, and sex. ^***b***^Model 1 plus adjusted for age, year of enrollment, sex, marital status, education level, occupation status, smoking status, years of smoking (among ever smokers), cigarettes/day (among current smokers). ^***c***^Model 2 plus adjusted for area-level variable {DCH: mean income at municipality level (16 units, median population ~ 1,500 inhabitants), per 100,000; EPIC-Turin, SIDRIA-Turin, and SIDRIA-Rome: deprivation index, census-block level (average population ~ 500 inhabitants); FINRISK: median income rate in a 3 x 3 km grid; HNR: unemployment rate, neighborhood level; KORA: percentage of low income in 5 x 5 km grid; SALT and SDPP: mean income in four categories, at municipality levels (area widths ranging from 9 km^2^ to 5,870 km^2^); SNAC-K: mean income in tertiles, at small neighborhoods level [small areas for market statistics (SAMS) based on election districts or similar, from Statistics Sweden (Stockholm and Örebro, Sweden)]; 60y: mean income in quartiles, at small neighborhoods level (SAMS, from Statistics Sweden)}. ^***d***^All except HNR. *p_heterogeneity_ < 0.05, as indicated by Cochran’s *Q* or *I*^2^ > 50%.						

The main PM_2.5_ results were robust to confounding adjustment and to model specification as found in extensive sensitivity analyses (Table 4). There were no relevant departures from the results of the main model after adjusting for intermediate variables, additional cardiovascular risk factors, and “rural” indicator or noise coexposure, or when stratified Cox models were implemented on the predictors that did not meet the PH assumption. Also, the results did not change when spatial autocorrelation was accounted for with “frailty” models (data not shown). We found marked differences in the PM_2.5_ associations with incident stroke depending on the precision of the cohort-specific LUR models in predicting individual PM_2.5_, with a significant estimate in the six cohorts with LOOCV *R^2^* coefficients > 0.6 (HR = 1.75; 95% CI: 1.30, 2.35), and no association in the other five cohorts (HR = 0.89; 95% CI: 0.70, 1.13).

**Table 4:** Association between PM_2.5_ exposure and stroke incidence in the 11 cohorts under study: results of the sensitivity analyses.

Model	Cohorts (*n*)	Participants (*n*)	HR (95% CI)
Main model	11	99,446	1.19 (0.88, 1.62)*
Role of cardiovascular risk factors
Intermediate variables: diabetes and hypertension
Plus diabetes and hypertension	11	99,446	1.15 (0.84, 1.56)*
Physical activity, alcohol consumption, and BMI
Main model, on subset of participants with additional information	8^*a*^	76,599	1.32 (0.87, 2.00)*
Plus additional information	8^*a*^	76,599	1.30 (0.86, 1.97)*
All cardiovascular risk factors (diabetes, hypertension, physical activity, alcohol, BMI, cholesterol)^*b*^
Main model, on subset of participants with additional information	4^*b*^	24,948	1.91 (0.96, 3.82)*
Plus additional information	4^*b*^	24,948	1.88 (0.99, 3.57)*
Urban/rural
Plus rural indicator	11	99,446	1.18 (0.87, 1.59)
Noise
Main model, on subset of participants with additional information	9^*c*^	73,121	1.25 (0.92, 1.71)
Plus noise variable	9^*c*^	73,121	1.26 (0.89, 1.78)
Change of address during follow-up
Main model, on cohorts with change of address data	10^*d*^	92,216	1.26 (0.93, 1.72)
No change of address during follow-up	10^*d*^	62,799	1.19 (0.81, 1.76)
Proportionality-hazards assumption
Variables which don’t meet PH assumption as strata	11	99,446	1.20 (0.89, 1.62)
Exclusion of DCH cohort
10 cohorts (all except DCH)	10	63,753	1.22 (0.86, 1.75)
Performance of the LUR model
LOOCV *R*^2^ coefficient > 0.6	6^*e*^	32,191	1.75 (1.30, 2.35)
LOOCV *R*^2^ coefficient ≤ 0.6	5^*f*^	67,255	0.89 (0.70, 1.13)
^***a***^All cohorts except SALT, SIDRIA-Turin and SIDRIA-Rome. ^***b***^Includes FINRISK, 60y, HNR, and KORA. ^***c***^All cohorts except SDPP and SIDRIA-Rome. ^***d***^All cohorts except EPIC-Turin. ^***e***^Includes SNAC-K (LOOCV *R*^2^ = 0.78), SALT (0.78), 60y (0.78), SDPP (0.78), HNR (0.79), and KORA (0.62). ^***f***^Includes FINRISK (LOOCV = *R*^2^ 0.53), DCH (0.55), EPIC-Turin (0.59), SIDRIA-Turin (0.59), and SIDRIA-Rome (0.60). *p_heterogeneity_ < 0.05, as indicated by Cochran’s *Q* or *I*^2^ > 50%.			

**Figure 1:**
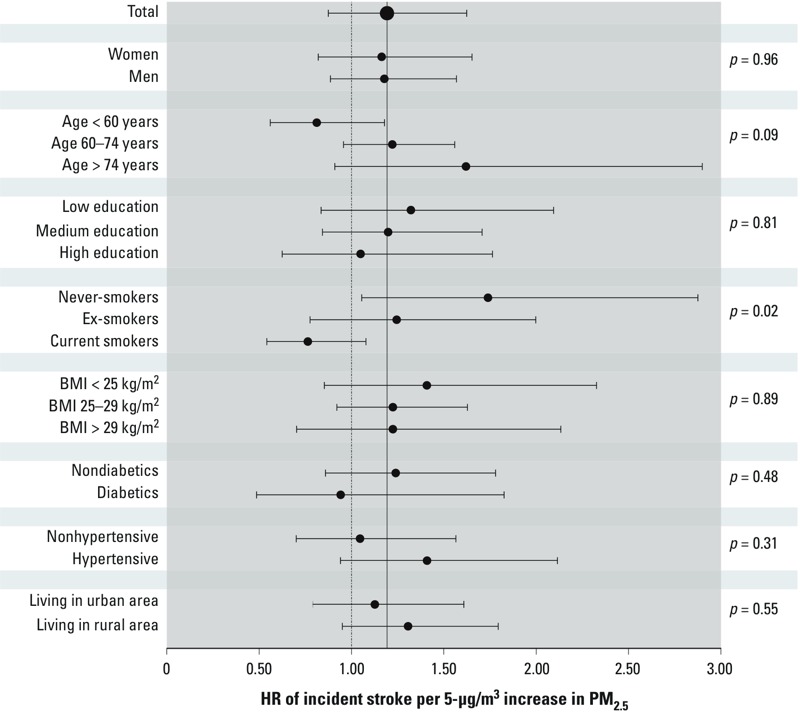
Association between PM_2.5_ exposure and stroke incidence in the 11 cohorts under study: results of the effect modification analysis. Values are HRs and 95% CIs per 5-μg/m^3^ increases in PM_2.5_. *p*-Values of effect modification (right) were calculated as heterogeneity tests among coefficients in different strata of the effect modifiers.

The results of the effect modification analysis are reported in Figure 1, together with the *p*-values for heterogeneity across the pooled strata-specific estimates. There was a suggestion of effect modification by age (*p* = 0.09), with a null effect at < 60 years (563 cases; HR = 0.81; 95% CI: 0.81, 1.18) and higher effects in the 60- to 74-year range (1,960 cases; HR = 1.22; 95% CI: 0.93, 1.61) and ≥ 75 years (563 cases; HR = 1.62; 95% CI: 0.91, 2.90) categories. The hazard ratio for those ≥ 60 years was 1.40 (95% CI: 1.05, 1.87) with little evidence of heterogeneity in the cohort-specific estimates (*I*^2^ = 25.8%; *p*_heterogeneity_ = 0.20, data not shown). Never-smokers had a significantly higher estimate of PM_2.5_ on the risk of incident stroke, with an increased risk of 74% (HR = 1.74; 95% CI: 1.06, 2.88).

The results from threshold models are reported in Table 5. We chose three exposure thresholds *a priori*: 25 μg/m^3^ (the current air quality limit value for annual average PM_2.5_ concentration in Europe), 20 μg/m^3^, and 15 μg/m^3^. The association between PM_2.5_ < 20 μg/m^3^ and incident stroke was high and borderline significant (HR = 1.29; 95% CI: 1.00, 1.68) for the nine cohorts with individuals below such concentrations. For the seven cohorts with PM_2.5_ concentrations below all the chosen thresholds, there was a 33% increased risk of incident stroke (HR = 1.33; 95% CI: 1.01, 1.77) for each 5-μg/m^3^ increase in PM_2.5_. The comparison of the linear and spline models suggested the linear shape of the concentration–response function as a good approximation for most of the cohorts (data not shown).

**Table 5:** Association between PM_2.5_ exposure and stroke incidence in subsets of the 11 cohorts under study: results of the threshold analyses.

Threshold (μg/m^3^)	Cohorts (*n*)	Participants (*n*)	HR (95% CI)
Cohorts with PM_2.5_ concentrations for the respective threshold
< 15	7^*a*^	72,769	1.24 (0.98, 1.58)
< 20	9^*b*^	84,496	1.29 (1.00, 1.68)
< 25	9^*b*^	86,812	1.29 (0.84, 1.98)*
Cohorts with PM_2.5_ concentrations available for all thresholds
Full range of exposure	7^*a*^	73,446	1.33 (1.01, 1.77)
< 15	7^*a*^	72,769	1.24 (0.98, 1.58)
< 20	7^*a*^	73,446	1.33 (1.01, 1.77)
< 25	7^*a*^	73,446	1.33 (1.01, 1.77)
^***a***^All except HNR, EPIC-Turin, SIDRIA-Turin and SIDRIA-Rome. ^***b***^All except EPIC-Turin and SIDRIA-Turin. *p_heterogeneity_ < 0.05, as indicated by Cochran’s *Q* or *I*^2^ > 50%.			

## Discussion

In this first multicenter European study on long-term exposure to ambient air pollution and stroke incidence, we found suggestive evidence of an association between PM_2.5_ exposure and stroke incidence, although the main estimate did not reach statistical significance. The results were robust to confounding adjustment and model specification. Stronger associations were estimated among participants ≥ 60 years old, never-smokers, and when all participants exposed to PM_2.5_ concentrations > 20 μg/m^3^ were removed from the analysis.

Most of the evidence on the effects of air pollution on stroke comes from time-series studies of cerebrovascular or stroke mortality (Maynard et al. 2007; O’Donnell et al. 2011; Wellenius et al. 2012). Evidence from previous studies on long-term effects is conflicting (Maaten and Brook 2011). The Women’s Health Initiative cohort study (Miller et al. 2007) found a 28% increased risk of stroke incidence in women (HR = 1.28; 95% CI: 1.02, 1.61) per 10-μg/m^3^ increase in PM_2.5_, which is similar to the estimate of the present study, 38% excess risk for 10-μg/m^3^ PM_2.5_ increments. In contrast, previous analyses of the American Cancer Society cohort (Krewski et al. 2009) and of a Norwegian cohort (Nafstad et al. 2004) failed to identify effects of air pollution on stroke mortality. More recently, a large prospective study conducted within the DCH cohort (which also contributes to the present analysis) detected a borderline significant association between NO_2_ and incident stroke (HR = 1.05; 95% CI: 0.99, 1.11, per 5.7-μg/m^3^ increase in NO_2_) (Andersen et al. 2012). In the present study, there was no association between long-term NO_2_ exposure and incident stroke in the DCH cohort. It should be considered that there are differences in the results reported from the DCH cohort: First, the whole cohort including the two largest cities in Denmark, Aarhus, and Copenhagen, contributed to the previous analysis, for a total of 52,215 participants, whereas only the Copenhagen part of the cohort was included in the present study (36,215 participants); second, the exposure assessment was different, because a dispersion model was used in the first analysis, with NO_2_ exposure assessed all the way back to 1971 and the mean from 1971 until the end of follow-up was used. A recent study in the United Kingdom found no relationship between long-term air pollution exposure and stroke incidence (Atkinson et al. 2013).

We noted significant heterogeneity in association estimates for most exposures. With the exception of the KORA (German Cooperative Health Research in the Region of Augsburg) cohort, all of the younger cohorts had point estimates for HRs, although nonsignificant, ≤ 1. When we restricted the study population to those ≥ 60 years of age, in light of the results of effect modification analyses suggesting the lowest risk is for those < 60 years of age and the highest for those > 74 years of age, we found that the heterogeneity was reduced (from *I*^2^ = 49.2%, *p*_heterogeneity_ = 0.032 for all ages, to *I*^2^ = 25.8%, *p*_heterogeneity_ = 0.20) and the increased relative risk was borderline statistically significant. Therefore, the different age composition of the cohorts seems to be the most plausible interpretation for the heterogeneity. However, it should be considered that a correlation was present between age and various characteristics of the cohorts (prevalence of smoking, quality of the LUR models, levels of air pollution exposures, and quality of the outcome assessment) with older cohorts having lower smoking rates, higher LUR LOOCV *R^2^* coefficients, lower PM_2.5_ levels, and stroke ascertainment based on expert medical record review in addition to mortality and hospitalization databases. It is therefore likely that any combination of these factors, not only age, might have been responsible for the heterogeneity in the associations across cohorts that we found. In addition, this is the most likely explanation for the stronger associations at lower exposure levels (< 20 μg/m^3^ PM_2.5_), for the cohorts with the highest LOOCV *R^2^* coefficients, better case ascertainment, and for the result among never-smokers.

In any case, the result for never-smokers is relevant because it indicates limited possibility of residual confounding from smoking and that the relative effect of ambient air pollution on stroke incidence is more easily detectable in the absence of a strong risk factor for stroke, such as active smoking.

A few limitations of the present study should be mentioned. First, air pollution measurement campaigns were implemented between 2008 and 2011, after the follow-up period of most cohorts (Cyrys et al. 2012; Eeftens et al. 2012b). As a consequence, this study relies on the assumption that the intracohort spatial distribution of air pollution has not dramatically changed in the last 10–15 years and that the land-use model predictions are thus representative of the baseline spatial contrasts for all the cohorts investigated. Several studies in the literature support this assumption over periods of about 10 years (Cesaroni et al. 2012; Eeftens et al. 2011). In addition, within the ESCAPE project many efforts were made to back-extrapolate air pollution concentrations, taking into account long-term time trends (see Supplemental Material, “Methods,” pp. 3–13), and analyses relating back-extrapolated data to the health outcomes showed no clear differences in the results compared with original data (data not shown). We also performed an exploratory analysis to evaluate whether the association between PM_2.5_ and stroke incidence differed according to accrual time, under the hypothesis that the assumption of stable spatial distribution of air pollution over time could be more valid for more recent cohorts: We did not find meaningful differences in the association estimates across cohorts according to accrual time (data not shown). Second, our approach exploited only within-study area contrasts, which limited the exposure contrast, but decreased the risk of potential confounding when comparing diverse cohorts from different countries. Third, the data available to adjust for confounding were somewhat different from cohort to cohort, allowing the possibility of different degrees of residual confounding in the cohort-specific results. However, the most relevant cardiovascular risk factors (smoking, diabetes or hypertension, BMI, physical activity) were available in almost all the cohorts, and thus severe bias in the effect estimates due to residual confounding is unlikely. Finally, we did not consider the possible impact of loss to follow-up (drop out or death) on the findings. Air pollution exposure is an established cause of mortality, so that older participants are likely to represent a population that is “selected” such that those who sustained higher exposures are more likely to have characteristics (genetic or otherwise) that place them at lower risk for stroke, resulting in underestimation of the causal relation of exposure with stroke risk. However, given the small relative risk of the air pollution–mortality association (i.e., HR < 1.10 for 5-μg/m^3^ increase in PM_2.5_ or 10-μg/m^3^ increase in PM_10_ or NO_2_ as reported by Beelen et al. 2013b), this underestimation is likely to be small.

This study has several strengths. The exposure assessment, one of the most critical aspects of this kind of study, was performed in a rigorous way with standardized procedures across all study areas (Cyrys et al. 2012; Eeftens et al. 2012b). The LOOCV *R^2^* coefficients from the PM_2.5_ LUR models ranged from 0.53 in Finland to 0.79 in Germany (Ruhr area), denoting strong discriminatory power of the spatial attributes used in these models to capture the spatial contrasts of exposures within the study areas (Eeftens et al. 2012a). An additional point of merit is the extensive list of variables available for confounding adjustment, including cardiovascular mediators and confounders, road traffic noise exposure at each residence and the urban/rural indicator used to characterize the degree of urbanization of each study area. Also, the statistical modeling was rigorously standardized between cohorts and addressed several methodological issues, including the potential for spatial autocorrelation of the study outcomes, and the linearity of the relationship between long-term air pollution exposure and stroke incidence.

## Conclusions

In summary, we found suggestive evidence of an association between long-term exposure to fine particles and stroke incidence in 11 European cohorts, especially among participants ≥ 60 years of age and among never-smokers. The association was also observed below current European limit values, indicating harmful effects of fine particles even at low concentrations.

## Supplemental Material

(1.2 MB) PDF
