# Supplementary material for: Long-Term Exposure to Ambient Air Pollution and Incidence of Cerebrovascular Events: Results from 11 European Cohorts within the ESCAPE Project
Source: Environ Health Perspect. 2014 May 16;122(9):919–25. doi: 10.1289/ehp.1307301 (PMC4153743; doi:10.1289/ehp.1307301)
Supplement: (1.2 MB) PDF [file ehp.1307301.s001.pdf]

Supplemental Material

Long-Term Exposure to Ambient Air Pollution and Incidence of Cerebrovascular Events: Results from 11 European Cohorts within the ESCAPE Project

Massimo Stafoggia, Giulia Cesaroni, Annette Peters, Zorana J. Andersen, Chiara Badaloni, Rob Beelen, Barbara Caracciolo, Josef Cyrys, Ulf de Faire, Kees de Hoogh, Kirsten T. Eriksen, Laura Fratiglioni, Claudia Galassi, Bruna Gigante, Aki S. Havulinna, Frauke Hennig, Agneta Hilding, Gerard Hoek, Barbara Hoffmann, Danny Houthuijs, Michal Korek, Timo Lanki, Karin Leander, Patrik K. Magnusson, Christa Meisinger, Enrica Migliore, Kim Overvad, Claes-Göran Östenson, Nancy L. Pedersen, Juha Pekkanen, Johanna Penell, Goran Pershagen, Noreen Pundt, Andrei Pyko, Ole Raaschou-Nielsen, Andrea Ranzi, Fulvio Ricceri, Carlotta Sacerdote, Wim J.R. Swart, Anu W. Turunen, Paolo Vineis, Christian Weimar, Gudrun Weinmayr, Kathrin Wolf, Bert Brunekreef, and Francesco Forastiere

Table of Contents **Page**

Methods **3**

Cohort details *3*

Outcome definition *10*

Prevalent cases *11*

Exposure assessment *11*

References **14**

Table S1. Study population: additional individual baseline characteristics [n (%)] **17**

Table S2. R^2 of LUR models[a]: model R^2 and leave-one-out cross-validation R^2 in the 11 cohorts **19**

Table S3. Predicted $PM_{2.5}$ concentrations[a] (μg/m^3): distribution in the 11 cohorts **20**

Figure S1. Map of the study area **21**

Figure S2. Forest plots of the cohort-specific results from the "base" model, with regard to the association between $PM_{2.5}$ and PM_{10} with stroke incidence (also, pooled estimates from random-effects meta-analysis are reported) **22**

Methods

Cohort details

- FINRISK: The National "FINRISK" surveys (Finalnd) have been conducted every 5 years since 1972 to monitor the risk factor trends of chronic diseases, including cardiovascular diseases, diabetes, cancer, asthma, and allergy (Peltonen et al. 2008). For each survey, a stratified random sample has been selected from the 25-64 (74 since 1997) year old inhabitants in different regions of Finland. The ESCAPE study used FINRISK data from four surveys (1992, 1997, 2002, 2007) and two study regions (the cities of Helsinki and Vantaa, and Turku city with its nearby municipalities). The FINRISK study protocol has been described elsewhere (Vartiainen et al. 2010). The surveys included a self-administered questionnaire (the questions focus mainly on socioeconomic factors, medical history, health behavior, and psychosocial factors) and a clinical examination including measurements of height, weight and blood pressure and blood sampling. The participants have been annually followed up through 31 Dec 2008 (up to 16 years) for fatal and nonfatal coronary and stroke events and total mortality. The National Hospital Discharge Register and the National Causes of Death Register were used to identify these events. These registers cover every hospitalization in Finland and every death of permanent residents in Finland, yielding in practice 100% coverage of the follow-up events (Pajunen at el. 2005; Tolonen et al. 2007). In addition, we used the drug reimbursement records from the Social Insurance Institution of Finland to identify subjects who had developed diabetes or hypertension during the follow-up period.
- SNAC-K: The Swedish National study of Aging and Care in Kungsholmen (SNAC-K) is an ongoing longitudinal study aiming to investigate the ageing process and identify possible preventive strategies to improve health and care in elderly adults (Lagergren et al. 2004). The

study population consists of randomly sampled individuals >=60 years old and in a central area of Stockholm (Kungsholmen) between March 2001 and June 2004. The sample was stratified for age and year of assessment giving sub-cohorts with 60, 66, 72, 78, 81, 84, 87, 90, 93, 96, and 99+ year olds. Information was collected through social interviews, assessment of physical functioning, clinical examination (incl. geriatric, neurological and physical assessments) as well as cognitive assessment. At baseline, information regarding events prior to the study period was gathered. The follow-up interval is six years for the younger age cohorts, and three years for the older age cohorts (81+). During the follow-up intervals, medical events of all subjects are registered through linkage with primary care registry and hospital discharge registry (available for all subjects in Sweden). In case of death, hospital and cause of death registries provide the clinical information, and informant interviews are carried out. The same protocol as for the baseline data collection is used during the follow-up, though only concerning the follow-up period. Website of study: http://www.aldrecentrum.se/snack/index.htm. Any outcomes based on the Swedish nationwide health registries (such as the myocardial infarction and stroke registries, the cause-of-death register and the national patient register) have been used.

- SALT: The Screening Across the Lifespan Twin Study (SALT) study was set-up to screen all twins born in Sweden before 1958 for the most common complex diseases with a focus on cardiovascular diseases (Lichtenstein et al. 2002; Lichtenstein et al. 2006). Twin Gene is a sub-study involving establishing a biobank with DNA and serum from SALT participants. SALT is based on a telephone interview, involving twins that were living in Stockholm County within the “Swedish national Twin Registry”. Recruitment took place between 1998-2002. Information concerning birth order and weight, zygosity, contact with twin partner and

family constellation, diseases, use of medication, occupation, education, life style habits, gender- and age-specific (hormone replacement therapy) and memory problems (age > 65) was collected. In Twin Gene, twins born before 1958 were contacted 2004-2008, a total number of ~2,500 participants was available. Health and medication data were collected from questionnaires. Blood sampling material was mailed to study subjects, who contacted a local health care centre for blood sampling and a health check-up. Height, weight, circumference of waist and hip, and blood pressure was measured and blood was collected. Any outcomes based on the Swedish nationwide health registries (such as the myocardial infarction and stroke registries, the cause-of-death register and the national patient register) have been used.

- 60y: The 60 year olds cohort is a study aiming to identify biological and socio-economic risk factors and predictors for cardiovascular diseases (Wändell et al. 2007). Recruitment took place between August 1997 and March 1999. A random sample of every third man and woman living in Stockholm County, who was born between 1 July 1937 and 30 June 1938, was invited to the 60 year olds study. In total 4232 subjects were included. Height, weight, BMI, Waist/Hip ratio and resting ECD, blood pressure and fasting blood samples were taken during a physical examination, while a comprehensive questionnaire was completed, including information on socioeconomic, medical and life-style factors. Any outcomes based on the Swedish national cause of death register and the patient register have been used.
- SDPP: The Stockholm Diabetes Preventive Program (SDPP), a population-based prospective study, aimed at investigating the aetiology of type 2 diabetes and developing prevention strategies for type 2 diabetes (Eriksson et al. 2008). An initial survey included all men and women in the targeted age group in Stockholm County; for men in four municipalities (Värmdö, Upplands Bro, Tyresö and Sigtuna), and for women these four plus a fifth

municipality (Upplands Väsby). All were screened by a questionnaire regarding presence of own diabetes and diabetes in relatives. Subjects with family history of diabetes (FHD) and randomly selected subjects without FHD, all without previously diagnosed diabetes, were invited to a health examination. This baseline study, 1992-1994 for men and 1996-1998 for women, comprised 7949 subjects, aged 35-56 years, and about 50% had FHD. In the follow-up study 8-10 years later, 2,383 men (2002-2004) and 3,329 women (2004-2006) participated. At the health examinations, both at baseline and follow-up, an extensive questionnaire (information on lifestyle factors, such as physical activity, dietary habits, tobacco use, alcohol consumption, health status, socioeconomic status and psychosocial conditions) was completed. Diabetes heredity was confirmed and measurements of weight, height, hip and waist circumference as well as blood pressure were performed. In addition, an oral glucose tolerance test (OGTT) was made, and blood was sampled at fasting state and 2 hour after glucose intake. Outcomes based on the Swedish nationwide health registries (such as the myocardial infarction and stroke registries, the cause-of-death register and the national patient register) have been used.

- DCH: The primary aim of the Danish Diet, Cancer, and Health (DCH) study is to investigate diet and lifestyle in relation to incidence of cancer and other chronic diseases (Tjønneland et al. 2007). The study combines the collection of questionnaire data with storing of biological specimen in order to investigate genetic susceptibility and gene-environment interactions with regard to diet, dietary compounds, and the risk of cancer, and endogenous markers of nutritional, metabolic, and hormonal characteristics of study participants. Historical residential history of the study participants is available, which facilitate studies of air pollution and noise. The study enrolled participants in two areas, Copenhagen and Aarhus,

Denmark. 160,725 individuals aged 50-64 years were invited to participate between December 1993 and May 1997. All participants were Danish-born, living in the Copenhagen or Aarhus areas and without medical history of cancer diagnosis registered in the Danish Cancer Registry at the time of invitation. Out of the 160,725 people invited, which were a random sample of all eligible individuals in the specified areas, 57,053 were enrolled. Due to the geographical limitations of the land use regression, only the almost 40,000 participants from the Copenhagen area were included in the ESCAPE analyses. On enrolment, each participant completed self-administered questionnaires (in Danish) that included questions on dietary habits, health status, family history of cancer, social factors, reproductive factors, smoking, environmental smoking and lifestyle habits. Anthropometric measurements including blood pressure and blood samples were also obtained. The DCH cohort is followed up regularly by use of complete nationwide registers hence the loss to follow-up is virtually nil.

- HNR: The Heinz Nixdorf Recall (HNR) study is an on-going population-based prospective cohort study, including 4,814 randomly selected participants 45 to 75 years of age at baseline (2000-2003) from three large adjacent cities (Essen, Mülheim and Bochum) of the densely and highly industrialized Ruhr area in western Germany (Schmermund et al. 2002). The HNR study was initiated to evaluate predictive value of EBCT compared to traditional and new risk factors in order to develop more effective methods of predicting cardiovascular disease in the general population. The study was approved by the institutional ethics committees and follows strict internal and external quality assurance protocols. Examination assessment included a self-administered questionnaire, face-to-face interviews for personal risk factor assessment, clinical examinations, and comprehensive laboratory tests according to standard

protocols. With regard to the ascertainment of stroke incident and prevalent cases, the HNR cohort relied on death certificates, and conducted an expert review of medical records by an independent endpoint committee (adjudication of cases) to ascertain cases during follow-up. Study design has been described in detail elsewhere (Schmermund et al. 2002).

- KORA: In the framework of the Cooperative Health Research in the Region of Augsburg (KORA), two cross-sectional population-representative surveys were conducted in 1994-1995 (S3 survey) and 1999-2001 (survey S4) in the city of Augsburg and two adjacent rural counties (Holle et al. 2005). The S3 survey was part of the WHO MONICA study (monitoring of trends and determinants in cardiovascular disease). Follow-up was carried out in 2008/2009. Main objective of the baseline investigation was to assess health indicators (morbidity, mortality) and health care (utilization, costs), to quantify the prevalence of risk factors of cardiovascular and other chronic diseases, and to study the impact of lifestyle, metabolic and genetic factors on cardiovascular and other chronic diseases. Sampling included all inhabitants of the Augsburg region of German nationality aged 25 to 74 (n=400,000). For each of the surveys, 6,640 inhabitants of the Augsburg region were randomly drawn, resulting in 4,856 (S3) and 4,261 (S4) participants, respectively (Response: 73% and 64%). Baseline examination included standardized interviews, physical examination, and blood sampling. Survival was ascertained for S3 participants in 2008 through Population Registry search and is available from the time of recruitment until 31.12.2007. Survival of S4 participants was ascertained through a combination of returned questionnaires and subsequent Population Registry search and is available from recruitment until 31.12.2008. Causes of death are recorded for all deaths from the death certificates. Morbidity follow up (incident coronary events) was tracked via questionnaires at follow up examinations, in questionnaires

sent by mail (2002 for F3, 2008 for F3/F4). self-reported incident cases of stroke and the date of diagnosis were validated by hospital records or by contacting the probands treating physician. Furthermore, the hospital records of those deceased during the follow-up period without a diagnosis of stroke at baseline were also examined and/or their last treating physicians were contacted. The records were searched for or the physicians were asked for a history of stroke and if a person had suffered from stroke, the type of stroke and the date of diagnosis were ascertained.

- EPIC: The European Prospective Investigation into Cancer and Nutrition (EPIC), which covers a large cohort of half a million men and women from 23 European centers in 10 Western European countries, was designed to study the relationship between diet and the risk of chronic diseases, particularly cancer (Riboli et al. 2002). 1 center from Turin, Italy, participated to this analysis (Guarrera et al. 2012). In EPIC-Turin, recruitment took place from 1993 and involved blood donors and other healthy volunteers, accruing 10604 participants by 1998. Co-operation with the local cancer registry and the local health authority allows for access to hospital discharge information and all newly diagnosed cancer cases. Follow-up started in 1998, including collaboration with the local cancer registry, the demographic computerized archives of the Torino area and the discharge report database for hospital patients.
- SIDRIA: The Italian Study on Respiratory Disorders in Childhood and Environment (SIDRIA) has been an extension of the ISAAC initiative in Italy (International Study on Asthma and Allergies in Childhood), a worldwide survey to analyze variations in prevalence of symptoms asthma, rhinitis, and atopic eczema. A cross-sectional survey was carried out between October 1994 and March 1995 in eight centres in northern and central Italy using

standardised questionnaires (response rate=94%). Parents of first and second graders from a representative sample of primary schools, and adolescents in the third year of a representative sample of junior high schools answered a self-administered questionnaire on the child's health status, as well as their personal respiratory health status and various risk factors, including education, occupation, housing conditions, smoking habits, and traffic intensity in their area of residence (SIDRIA 1997). The data used within ESCAPE are from the subset of parents recruited in two metropolitan areas: Rome and Turin, in the context of a project co-funded by the Ministry of Health (Programma Strategico Ambiente e Salute, Ricerca Finalizzata exart. 12, 2006) (Cesaroni et al. 2008). A record linkage has been performed with the Municipal Registry Office Databases to collect the residential history of parents who were living in Rome and Turin with their children at the time of the survey. In the city of Turin the project was performed through a collaboration between SIDRIA and the regional Unit of Epidemiology (ASL TO3), in the context of the Turin Longitudinal Study, a census-based cohort study following up health outcomes of people censused in Turin since 1971. It was possible to identify ~16,000 adults.

Outcome definition

The identification of first cerebrovascular events during follow-up was accomplished by interview and inspection of medical records and death certificates in two cohorts (HNR and KORA) out of 11 (Holle et al. 2005; Schmermund et al. 2002). Otherwise, the outcomes were identified by record-linkage of the individual records with mortality registries and hospital discharge databases, according to the following criteria based on the International Classification of Diseases (ICD) (WHO 1999), 9th and 10th revisions codes:

- hospitalizations with principal diagnosis of ischemic stroke (ICD9: 433.x1, 434; ICD10: I63), hemorrhagic stroke (ICD9: 431; ICD10: I61), unspecified stroke (ICD9: 436; ICD10: I64);
- out-of-hospital deaths from cerebrovascular diseases (ICD9: 431-436; ICD10: I61-I64).

Prevalent cases

Prevalent cases of either coronary or cerebrovascular disease at baseline were excluded. Methods to define and ascertain prevalent cases differed between the cohorts. For most of the cohorts, prevalent cases were defined by information collected at baseline by a questionnaire on acute myocardial infarction, stroke history and medication use. When only hospitalization data were available, at least three years of data before enrolment were considered. Prevalent cases of acute coronary events were defined as hospital admissions for an acute coronary event in the previous period (ICD9: 410-412; ICD10: I20.0, I25.2, I21, I23, I24). Similarly, prevalent cases of stroke were defined as hospital discharge in the previous three years with a diagnosis of stroke or late effects of cerebrovascular disease (ICD9: 431, 434, 433.x1, 436, 438; ICD10: I61, I63, I64, I69).

Exposure assessment

Long-term exposure to ambient air pollutants at the residential address of each individual was estimated within the ESCAPE project following a three-step procedure.

First, $PM_{2.5}$, $PM_{2.5}$ absorbance (a proxy for elemental carbon), PM_{10}, NO_2 and NO_x were measured between October 2008 and April 2011 in 20 European areas using standardized protocols (Cyrys et al. 2012; Eeftens et al. 2012). In each study area, 20 PM monitoring sites and 40 NO_x monitoring sites were chosen to represent the variability in important air quality predictors, such as population density, traffic intensity and altitude. Measurements were taken at each site over three 14-day periods in different seasons. Results for each location were averaged

after correcting for temporal variation using data obtained from a reference site, which was in operation year-round. Coarse PM was calculated as the difference between PM_{10} and $PM_{2.5}$.

Second, land-use regression models were developed for each study area and pollutant, with site-specific annual average concentrations as the dependent variables, and an extensive list of spatial attributes as predictors. These included: roads characteristics, land-use data, population-density data, altitude, etc. The indicators of model performance were generally good for all pollutants, with leave-one-out cross-validation (LOOCV) R-squared coefficients for $PM_{2.5}$ ranging from 0.53 (FINRISK, Finland) to 0.79 (HNR, Ruhr area).

Third, once the models were optimized, their coefficients were applied to predict annual air pollution averages at the residence address of each cohort member. If values of predictor variables for the cohort addresses were outside the range of values for the monitoring sites, values were truncated to the minimum and maximum values at the monitoring sites. Truncation was performed to prevent unrealistic predictions (e.g. related to too small distance to roads in GIS) and because we did not want to extrapolate the derived model beyond the range for which it was developed. Truncation has been shown to improve predictions at independent sites. The estimates so obtained were considered as the reference long-term exposures in the present study.

Pollution measurements were performed in 2008-2011, but follow-up from baseline addresses was in all cohorts covering earlier time periods. We therefore extrapolated predicted concentrations back in time using the absolute difference and the ratio between the two periods, based on data from routine background monitoring network site(s) in the study areas. Details on this procedure can be found on the website http://www.escapeproject.eu/manuals/.

In addition, two traffic variables were used as relevant exposures: the traffic intensity on the nearest road (measured as the number of vehicles/day), and the traffic load on major roads within a 100m buffer (measured as the product of the intensity and the length of the roads intersecting the buffer). These variables were evaluated alone, and in combination with predicted background NO_2 concentrations. The noise exposure assessment was carried out locally. More specifically, the noise level (Lden: day-evening-night equivalent level) was calculated for the most exposed façade of dwellings. National calculation methods were used in the study areas of the Finnish, Swedish, Danish (Nordic Prediction Method) and German cohorts. The interim method of the EU was applied for EPIC Turin and SIDRIA (WG-AEN 2007). Noise barriers and actual or estimated building heights and terrain effects from absorption and reflection in the ground were included in the modelling. To assess traffic flow, composition and speed for the full road network different methods were applied within each study area. For motorways actual traffic counts were used. For other roads, if counts were not available, traffic forecast models were used or flows were estimates from counts. For smaller road standard traffic flows were used. For roads were no actual composition was known, standard distributions were used. For the majority of the roads the speed limit was used. The exposure at the most exposed façade with a resolution of 0.1 dB was obtained from grids (FINRISK 10x10m, Stockholm city 5x5m, Stockholm county 25x25m, HNR 10x10 m, EPIC Turin and SIDRIA 5x5 m) or by using an assessment point directly at this façade (DCH, KORA). The software used was CadnaA (FINRISK, Stockholm county, HNR, KORA) and Soundplan (FINRISK, Stockholm city and county, DCH), and a general ArcGIS noise model (EPIC Turin, SIDRIA). First order reflections were included in the calculations.

References

Cesaroni G, Badaloni C, Porta D, Forastiere F, Perucci CA. 2008. Comparison between various indices of exposure to traffic-related air pollution and their impact on respiratory health in adults. Occup Environ Med 65:683-690.

Cyrys J, Eeftens M, Heinrich J, Ampe C, Armengaud A, Beelen R, et al. 2012. Variation of NO2 and NOx concentrations between and within 36 European study areas: Results from the ESCAPE study. Atmos Environ 62:374-390.

Eeftens M, Tsai M, Ampe C, Anwander B, Beelen R, Bellander T, et al. 2012. Spatial variation of PM2.5, PM10, PM2.5 absorbance and PM coarse concentrations between and within 20 European study areas and the relationship with NO2 - results of the ESCAPE project. Atmos Environ 62:303-317.

Eriksson AK, Ekbom A, Granath F, Hilding A, Efendic S, Ostenson CG. 2008. Psychological distress and risk of pre-diabetes and Type 2 diabetes in a prospective study of Swedish middle-aged men and women. Diabetic Med 25:834-842.

Guarrera S, Ricceri F, Polidoro S, Sacerdote C, Allione A, Rosa F, et al. 2012. Association between total number of deaths, diabetes mellitus, incident cancers, and haplotypes in chromosomal region 8q24 in a prospective study. Am J Epidemiol 175:479-487.

Holle R, Happich M, Löwel H, Wichmann HE; MONICA/KORA Study Group. 2005. KORA–a research platform for population based health research. Gesundheitswesen 67 Suppl 1:S19-S25. Review.

Lagergren M, Fratiglioni L, Hallberg IR, Berglund J, Elmståhl S, Hagberg B, et al. 2004. A longitudinal study integrating population, care and social services data. The Swedish National study on Aging and Care (SNAC). Aging Clin Exp Res 16:158-168.

Lichtenstein P, De Faire U, Floderus B, Svartengren M, Svedberg P, Pedersen NL. 2002. The Swedish Twin Registry: A unique resource for clinical, epidemiological and genetic studies. J Intern Med 252:184-205.

Lichtenstein P, Sullivan PF, Cnattingius S, Gatz M, Johansson S, Carlström E, et al. 2006. The Swedish Twin Registry in the Third Millennium - an update. Twin Res Hum Genet 9:875-882.

Pajunen P, Koukkunen H, Ketonen M, Jerkkola T, Immonen-Räihä P, Kärjä-Koskenkari P, et al. 2005. The validity of the Finnish Hospital Discharge Register and Causes of Death Register data on coronary heart disease. Eur J Cardiovasc Prev Rehabil 12:132-137.

Peltonen M, Harald K, Männistö S, Saarikoski L, Lund L, Sundvall J, et al. 2008. The National FINRISK 2007 Study. Publications of the National Public Health Institute, Report No.: B34/2008.

Riboli E, Hunt KJ, Slimani N, Ferrari P, Norat T, Fahey M, et al. 2002. European Prospective Investigation into Cancer and Nutrition (EPIC): Study populations and data collection. Public Health Nutr 5:1113-1124.

Schmermund A, Möhlenkamp S, Stang A, Grönemeyer D, Seibel R, Hirche H, et al. 2002. Assessment of clinically silent atherosclerotic disease and established and novel risk factors for predicting myocardial infarction and cardiac death in healthy middle-aged subjects: rationale and design of the Heinz Nixdorf RECALL study. Risk factors, evaluation of coronary calcium and lifestyle. Am Heart J 144:212-218.

SIDRIA. 1997. Asthma and respiratory symptoms in 6-7 yr old Italian children: gender, latitude, urbanization and socioeconomic factors. Eur Respir J 10:1780-1786.

Tjønneland A, Olsen A, Boll K, Stripp C, Christensen J, Engholm G, et al. 2007. Study design, exposure variables, and socioeconomic determinants of participation in Diet, Cancer and Health: A population-based prospective cohort study of 57,053 men and women in Denmark. Scand J Public Health 35:432-441.

Tolonen H, Salomaa V, Torppa J, Sivenius J, Immonen-Räihä P, Lehtonen A. 2007. The validation of the Finnish Hospital Discharge Register and Causes of Death Register data on stroke diagnoses. Eur J Cardiovasc Prev Rehabil 14:380-385.

Vartiainen E, Laatikainen T, Peltonen M, Juolevi A, Männistö S, Sundvall J, et al. 2010. Thirty-five-year trends in cardiovascular risk factors in Finland. Int J Epidemiol 39:504-518.

Wändell PE, Wajngot A, de Faire U, Hellenius ML. 2007. Increased prevalence of diabetes among immigrants from non-European countries in 60-year-old men and women in Sweden. Diabetes Metab 33:30-36.

WG-AEN (Working Group Assessment of Exposure to Noise). 2007. Good Practice Guide for Strategic Noise Mapping and the Production of Associated Data on Noise Exposure Position Paper. Version 2. http://ec.europa.eu/environment/noise/pdf/gpg2.pdf (Accessed January 11, 2013).

WHO (World Health Organization). 1999. International Classification of Diseases (ICD). Available: http://www.who.int/classifications/icd/en/ [accessed 27 June 2013].

Table S1. Study population: additional individual baseline characteristics [n (%)].

Individual characteristics	FINRISK	SNAC-K	Salt Twin G	60 y olds	SDPP	DCH	RECALL	KORA	EPIC-Turin	SIDRIA-Turin	SIDRIA-Rome
Area-level socio-economic variable[a]											
1	22,946 ± 5,458	1,429 (53.2)	1,692 (27.8)	898 (24.4)	3,024 (39.2)	1.9 ± 0.4	12.6 ± 3.1	28.0 ± 18.4	1,859 (25.7)	895 (17.4)	1,707 (18.6)
2	-	1,252 (46.6)	2,643 (43.4)	924 (25.1)	1,164 (15.1)	-	-	-	1,662 (23.0)	1,070 (20.8)	1,684 (18.3)
3	-	3 (0.1)	242 (4.0)	938 (25.4)	2,016 (26.1)	-	-	-	1,342 (18.6)	944 (18.4)	1,675 (18.2)
4	-	-	1,507 (24.8)	926 (25.1)	1,519 (19.7)	-	-	-	1,404 (19.4)	1,114 (21.7)	1,802 (19.6)
5	-	-	-	-	-	-	-	-	963 (13.3)	1,114 (21.7)	2,332 (25.3)
Mean ± SD body mass index	26.3 ± 4.6	25.6 ± 4.1	28.5 ± 4.1	26.8 ± 4.2	25.7 ± 4.0	26.0 ± 4.1	27.8 ± 4.6	27.0 ± 4.5	25.3 ± 3.8	-	-
Body mass index in classes											
BMI < 25	4,329 (43.3)	1,209 (48.1)	1,055 (17.6)	1,349 (36.6)	3,741 (48.6)	15,998 (44.9)	1,201 (27.2)	2,592 (34.5)	3,691 (51.0)	-	-
BMI between 25 and 29	3,852 (38.6)	1,004 (39.9)	3,131 (52.3)	1,633 (44.3)	2,995 (38.9)	14,642 (41.1)	2,025 (45.9)	3,272 (43.5)	2,779 (38.4)	-	-
BMI > 29	1,811 (18.1)	302 (12.0)	1,805 (30.1)	704 (19.1)	9,64 (12.5)	5,026 (14.1)	1,189 (26.9)	1,652 (22.0)	761 (10.5)	-	-
Physical activity											
Once a month or less /<1h/week	1,808 (18.1)	464 (20.5)	1,575 (26.0)	2,510 (68.8)	846 (11.0)	15,984 (44.8)	2,263 (51.2)	2,783 (36.8)	1,660 (23.0)	-	-
About once a week /~1h/week	3,322 (33.3)	686 (30.3)	3,757 (62.1)	858 (23.5)	6,273 (81.3)	10,890 (31.6)	490 (11.1)	3,237 (42.8)	2,032 (28.1)	-	-
3 times a week or more />2h/week	4,839 (48.5)	1,115 (49.2)	716 (11.8)	279 (7.7)	597 (7.7)	7,716 (22.4)	1,667 (37.7)	1,548 (20.5)	3,538 (48.9)	-	-
Alcohol consumption											
Never	1,320 (13.4)	519 (19.4)	-	168 (4.6)	594 (7.8)	776[b] (2.2)	1,015 (23.4)	3,501 (46.2)	457 (6.3)	-	-
1-3 drinks/week	4,566 (46.4)	634 (23.7)	-	771 (20.9)	2,876 (37.7)	24,589[b] (68.9)	1,190 (27.5)	1,113 (14.7)	201 (2.8)	-	-
3-6 drinks/week	3,139 (31.9)	1,236 (46.2)	-	1,622 (44.0)	3,873 (50.8)	7,707[b] (21.6)	2,127[c] (49.1)	511 (6.8)	347 (4.8)	-	-
>6 drinks/week	824 (8.4)	288 (10.8)	-	1,123 (30.5)	280 (3.7)	2,621[b] (7.3)	-	2,445 (32.3)	6,225 (86.1)	-	-
Diagnosed diabetes											
No	9,546 (95.7)	2,464 (91.8)	5,833 (95.9)	3,533 (95.8)	7,597 (98.4)	34,943 (98.0)	3,877 (87.5)	7,254 (95.7)	7,118 (98.5)	5,111 (99.5)	9,179 (99.8)
Yes	426 (4.3)	220 (8.2)	251 (4.1)	153 (4.2)	126 (1.6)	703 (2.0)	556 (12.5)	327 (4.3)	110 (1.5)	26 (0.5)	21 (0.2)

Individual characteristics	FINRISK	SNAC-K	Salt Twin G	60 y olds	SDPP	DCH	RECALL	KORA	EPIC-Turin	SIDRIA-Turin	SIDRIA-Rome
Diagnosed hypertension											
No	5,877 (59.2)	902 (33.9)	4,735 (77.9)	1,770 (48.0)	5,801 (75.9)	30,033 (84.2)	2,002 (45.2)	4,680 (61.8)	3,629 (52.7)	5,082 (98.9)	9,124 (99.2)
Yes	4,042 (40.8)	1,762 (66.1)	1,347 (22.1)	1,915 (52.0)	1,837 (24.1)	5,625 (15.8)	2,423 (54.8)	2,891 (38.2)	3,255 (47.3)	55 (1.1)	76 (0.8)
Mean ± SD cholesterol level	210.6 ± 77.7	-	-	231.1 ± 41.3	-	-	230.8 ± 38.9	229.1 ± 43.7	-	-	-
Noise exposure at residence address											
< 45 dB	-	16 (0.5)	123 (5.0)	84 (5.7)	-	126 (0.4)	721 (16.7)	427 (5.6)	44 (0.8)	63 (1.2)	-
45-49 dB	1,912[c] (22.9)	85 (2.6)	200 (8.2)	110 (7.4)	-	1,838 (5.2)	989 (23.0)	1,221 (16.1)	115 (2.0)	121 (2.4)	-
50-54 dB	2,040 (24.4)	26 (0.8)	524 (21.4)	292 (19.7)	-	9,634 (27.0)	853 (19.8)	2,710 (35.8)	87 (1.5)	103 (2.0)	-
55-59 dB	1,721 (20.6)	265 (8.0)	550 (22.5)	304 (20.6)	-	9,560 (26.8)	565 (13.1)	1,705 (22.5)	253 (4.5)	186 (3.6)	-
60-64 dB	1,181 (14.1)	1,010 (30.3)	435 (17.8)	291 (19.7)	-	7,625 (21.4)	482 (11.2)	903 (11.9)	2,419 (42.6)	2,237 (43.7)	-
65-69 dB	784 (9.4)	866 (26.0)	332 (13.6)	225 (15.2)	-	4,600 (12.9)	462 (10.7)	439 (5.8)	1,254 (22.1)	1,133 (22.1)	-
70-74 dB	714[c] (8.5)	766 (23.0)	237 (9.7)	143 (9.7)	-	2,010 (5.6)	194 (4.5)	171 (2.3)	1,369 (24.1)	1,126 (22.0)	-
> 74 dB	-	298 (8.9)	43 (1.8)	30 (2.0)	-	294 (0.8)	40 (0.9)	2 (0.0)	136 (2.4)	156 (3.0)	-
Living in rural area											
No	9,086 (90.9)	2,684 (100.0)	2,763 (45.4)	1,450 (39.3)	0 (0.0)	14,155 (39.7)	4,433 (100.0)	3,225 (42.5)	7,230 (100.0)	5,137 (100.0)	9,200 (100.0)
Yes	909 (9.1)	0 (0.0)	3,321 (54.6)	2,236 (60.7)	7,723 (100.0)	21,538 (60.3)	0 (0.0)	4,356 (57.5)	0 (0.0)	0 (0.0)	0 (0.0)

[a]Mean ± standard deviations are reported for continuous variables. FINRISK: median income rate in a 3x3 km grid; SNAC-K: mean income in tertiles, at small neighborhoods level (Small Areas for Market Statistics (SAMS) based on election districts or similar, from Statistics Sweden); SALT and SDPP: mean income in 4 categories, at municipality levels (area widths ranging from 9 km^2 to 5,870 km^2); 60y: mean income in quartiles, at small neighborhoods level (Small Areas for Market Statistics (SAMS) based on election districts or similar, from Statistics Sweden); DCH: mean income at municipality level (16 units, median population ~1500 inhabitants), per/100,000; HNR: unemployment rate, neighborhood level; KORA: percentage of low income in 5x5 km grid; EPIC-Turin, SIDRIA-Turin and SIDRIA-Rome: deprivation index, census-block level (average population ~ 500 inhabitants). [b]1 drink=10g alcohol. [c]The first / last two classes are collapsed.

Table S2. R^2 of LUR models[a]: model R^2 and leave-one-out cross-validation R^2 in the 11 cohorts.

Cohorts	$PM_{2.5}$	Coarse PM	PM_{10}	Absorbance $PM_{2.5}$	NO_2	NO_x
FINRISK	0.67-0.53	0.61-0.33	067-0.42	0.65-0.47	0.83-0.75	0.85-0.74
SNAC-K	0.87-0.78	0.72-0.65	0.82-0.77	0.89-0.85	0.82-0.78	0.83-0.79
SALT	0.87-0.78	0.72-0.65	0.82-0.77	0.89-0.85	0.82-0.78	0.83-0.79
60y	0.87-0.78	0.72-0.65	0.82-0.77	0.89-0.85	0.82-0.78	0.83-0.79
SDPP	0.87-0.78	0.72-0.65	0.82-0.77	0.89-0.85	0.82-0.78	0.83-0.79
DCH	0.62-0.55	0.71-0.54	0.75-0.64	0.92-0.86	0.88-0.83	0.83-0.73
HNR	0.88-0.79	0.66-0.57	0.69-0.63	0.97-0.95	0.89-0.84	0.88-0.81
KORA	0.78-0.62	0.81-0.79	0.83-0.75	0.91-0.82	0.86-0.67	0.88-0.76
EPIC-Turin	0.71-0.59	0.65-0.58	0.78-0.69	0.88-0.81	0.78-0.70	0.78-'0.72
SIDRIA-Turin	0.71-0.59	0.65-0.58	0.78-0.69	0.88-0.81	0.78-0.70	0.78-'0.72
SIDRIA-Rome	0.71-0.60	0.70-0.57	0.72-0.59	0.84-0.70	0.87-0.76	0.80-0.79

[a]For each cohort and pollutant, the R^2 coefficients of the land-use regression (LUR) models are reported: the first value is the model R^2, the second value is the leave-one-out cross-validation R^2 obtained by deleting one monitor at a time and computing the R^2 coefficient on the remaining ones.

Table S3. Predicted $PM_{2.5}$ concentrations[a] ($\mu g/m^3$): distribution in the 11 cohorts.

Cohorts	n	Mean	SD	Min	5th percentile	25th percentile	50th percentile	75th percentile	95th percentile	Max	IQR	5-95 range
FINRISK	9,995	7.7	1.1	5.6	5.6	7.0	7.9	8.6	9.1	11.0	1.6	3.5
SNAC-K	2,684	7.9	1.3	4.5	5.8	7.1	8.0	8.8	10.2	11.6	1.7	4.4
SALT	6,084	7.3	1.3	4.2	5.0	6.5	7.5	8.2	9.2	11.8	1.7	4.2
60y	3,686	7.3	1.3	4.2	4.9	6.4	7.4	8.1	9.2	11.9	1.8	4.3
SDPP	7,723	6.6	1.2	4.2	4.6	5.7	6.7	7.5	8.5	10.6	1.8	3.9
DCH	35,693	11.3	0.9	7.4	9.8	10.9	11.4	11.9	12.6	12.8	1.0	2.8
HNR	4,433	18.4	1.1	16.0	16.8	17.6	18.3	19.1	20.4	21.4	1.5	3.5
KORA	7,581	13.6	0.9	11.5	12.5	13.0	13.5	14.1	15.3	17.7	1.1	2.8
EPIC-Turin	7,230	30.1	1.7	21.4	26.9	29.4	30.3	30.9	32.6	35.6	1.5	5.7
SIDRIA-Turin	5,137	31.0	1.7	21.6	28.6	30.4	30.7	31.6	34.5	35.6	1.2	5.9
SIDRIA-Rome	9,200	19.4	1.8	17.0	17.3	18.2	18.9	20.0	23.3	27.4	1.7	6.0

[a]For each cohort, the distribution of the $PM_{2.5}$ concentrations predicted from the land-use regression models are reported: number of cohort participants, mean and standard deviation of the $PM_{2.5}$ concentrations predicted at the residence address, minimum, percentiles and maximum values, interquartile range (IQR, obtained as the difference between the 3rd and the 1st quartiles), and 5-95 range (obtained as the difference between the 95th and the 5th percentiles).

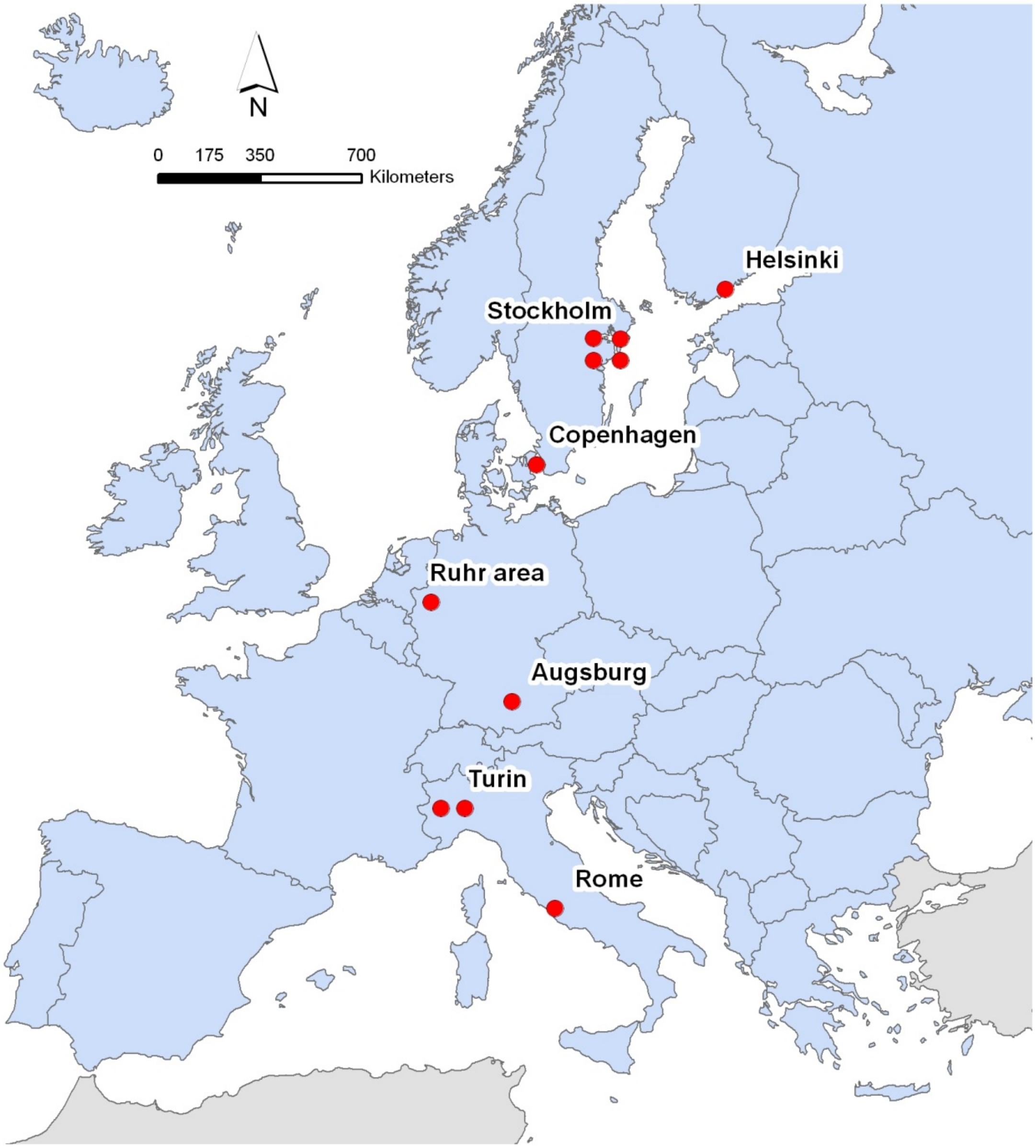

Figure S1. Map of the study area.

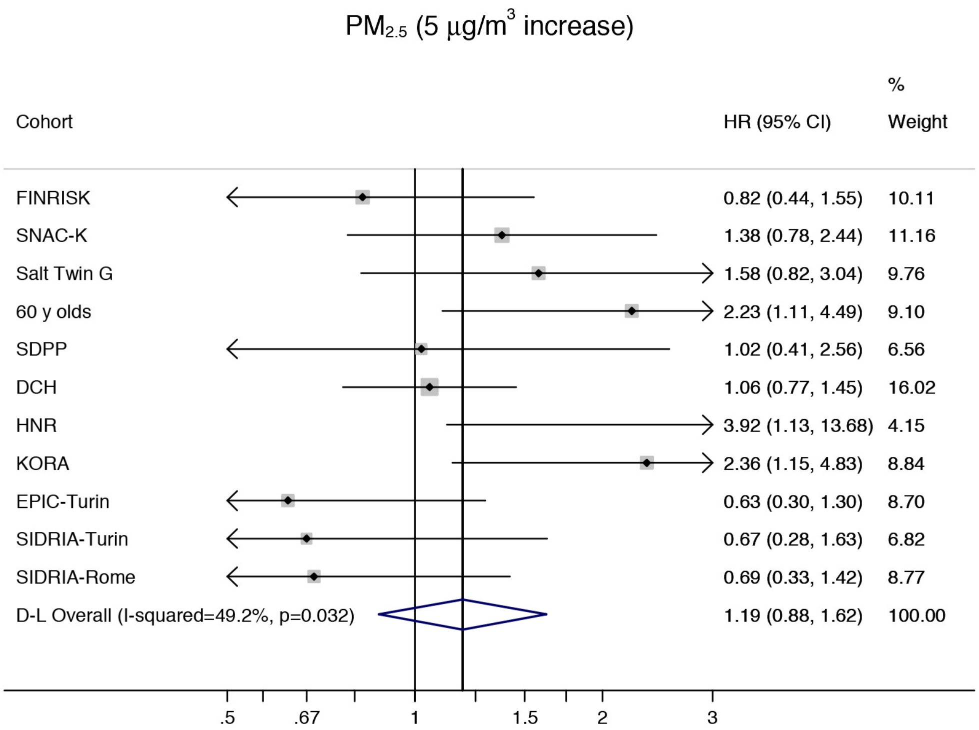

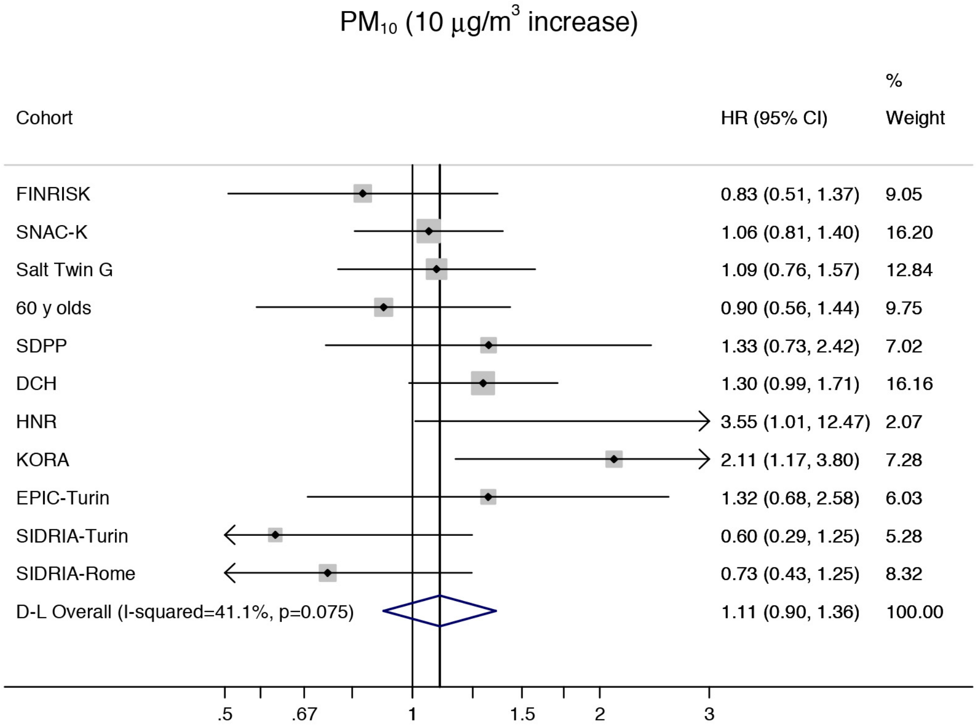

Figure S2. Forest plots of the cohort-specific results from the "base" model, with regard to the association between $PM_{2.5}$ and PM_{10} with stroke incidence (also, pooled estimates from random-effects meta-analysis are reported).
